# *Neisseria gonorrhoeae* Modulates Iron-Limiting Innate Immune Defenses in Macrophages

**DOI:** 10.1371/journal.pone.0087688

**Published:** 2014-01-28

**Authors:** Susu M. Zughaier, Justin L. Kandler, William M. Shafer

**Affiliations:** 1 Department of Microbiology and Immunology, Emory University School of Medicine, Atlanta, Georgia, United States of America; 2 Laboratories of Microbial Pathogenesis, Department of Veterans Affairs Medical Center, Decatur, Georgia, United States of America; Monash University, Australia

## Abstract

*Neisseria gonorrhoeae* is a strict human pathogen that causes the sexually transmitted infection termed gonorrhea. The gonococcus can survive extracellularly and intracellularly, but in both environments the bacteria must acquire iron from host proteins for survival. However, upon infection the host uses a defensive response by limiting the bioavailability of iron by a number of mechanisms including the enhanced expression of hepcidin, the master iron-regulating hormone, which reduces iron uptake from the gut and retains iron in macrophages. The host also secretes the antibacterial protein NGAL, which sequesters bacterial siderophores and therefore inhibits bacterial growth. To learn whether intracellular gonococci can subvert this defensive response, we examined expression of host genes that encode proteins involved in modulating levels of intracellular iron. We found that *N. gonorrhoeae* can survive in association (tightly adherent and intracellular) with monocytes and macrophages and upregulates a panel of its iron-responsive genes in this environment. We also found that gonococcal infection of human monocytes or murine macrophages resulted in the upregulation of hepcidin, NGAL, and NRAMP1 as well as downregulation of the expression of the gene encoding the short chain 3-hydroxybutyrate dehydrogenase (BDH2); BDH2 catalyzes the production of the mammalian siderophore 2,5-DHBA involved in chelating and detoxifying iron. Based on these findings, we propose that *N. gonorrhoeae* can subvert the iron-limiting innate immune defenses to facilitate iron acquisition and intracellular survival.

## Introduction

*Neisseria gonorrhoeae* is a strict human pathogen that causes the sexually transmitted disease gonorrhea with more than 100 million cases estimated yearly world-wide [1]. Gonococci can cause both symptomatic and asymptomatic infections in men and women, which is thought to be dictated by host and bacterial factors that determine the extent of stimulation of the pro-inflammatory response. Combined with the large number of infections worldwide and the medical complications associated with infection, particularly for women, the emergence of antibiotic-resistant strains of gonococci is now a major public health concern with worrisome predictions that gonorrhea may become an untreatable disease unless new antimicrobials are developed [2], [3], [4].

Gonococci express virulence factors that facilitate infection and promote survival within host phagocytic and epithelial cells [1]. Most work on such intracellular survival has concentrated on how the gonococcus subverts oxidative and nonoxidative killing systems of neutrophils [1], [5]. Much less is known, however, regarding how it acquires nutrients for growth, especially iron, during intracellular residence. In this respect, most iron, whether in the extracellular or intracellular environments, is tightly complexed with iron-binding proteins and not readily available for microbes. To circumvent this problem, bacteria can either remove and transport iron to their surface through the action of siderophores or use surface-exposed proteins that bind host proteins complexed with iron (e.g., transferrin, ferritin, lactoferrin, and hemoglobin) [6], [7], [8]. The host can also influence the ability of bacteria to acquire iron by secreting NGAL (neutrophil gelatinase-associated lipocalin), which sequesters bacterial siderophores [9], [10]. Additionally, the host increases production of hepcidin, the master iron-regulating hormone, to limit the bioavailability of iron [7]. This host defense strategy, called the iron-limiting innate immune defense [6], can influence iron availability and survival of intracellular bacteria in response to infection.

Macrophages play an important role in innate immunity and a central role in iron homeostasis. Since macrophages engulf senescent and damaged red blood cells (RBCs), they recycle iron daily in a process known as erythrophagocytosis [10]. Therefore, during infection, cellular iron metabolism is tightly regulated. A key cellular iron regulator involved in iron-limiting host defense is hepcidin [11], which is the master iron-regulating hormone that retains iron in macrophages [12] by binding to ferroportin (SLC40A1) [13], the only known iron exporter protein that exports iron to the extracellular milieu, leading to the internalization and subsequent degradation of ferroportin. Moreover, the action of NRAMP1 (Natural resistance-associated macrophage protein 1 or SLC11A1) [14] allows for transport of iron from the late endosome and phagolysosome to the cytosol where it can be safely stored in ferritin cages [15] or as part of iron-sulfur clusters. As a consequence, during infection both hepcidin and NRAMP1 increase the cytosolic labile iron pool in macrophages. Since free labile iron is toxic, the cytosolic enzyme 3-hydroxybutyrate dehydrogenase type 2 (BDH2) [16] detoxifies cytosolic iron by catalyzing the synthesis of the mammalian siderophore 2,5-DHBA that binds free iron. Thus, BDH2 is also required for cellular iron homeostasis [17]. Further, NGAL, the iron carrier protein that shuttles and delivers liganded iron for cellular growth and differentiation [18] and scavenges bacterial siderophores [9], thereby exerts antibacterial function [19].

The aim of this work was to investigate whether macrophages sense the presence of intracellular *N. gonorrhoeae* and whether gonococci evade or modulate the host iron-limiting innate immune defenses. We now report that *N. gonorrhoeae* can survive intracellularly or in association with monocytes and macrophages, and that gonococcal infection of these cells upregulates expression of hepcidin, NGAL, and NRAMP1 and downregulates BDH2 and ferroportin expression. We hypothesize that such modulation facilitates gonococcal iron acquisition by increasing the cellular labile iron pool and its bioavailability.

## Materials and Methods

### Reagents

RPMI1640 medium, Dulbecco's modified Eagle medium (D-MEM), fetal bovine serum (FBS), penicillin/streptomycin, sodium pyruvate and nonessential amino acids were obtained from Cellgro Mediatech (Herdon, VA). Human NGAL, TNFα, IL-1β, IL-6 and CXCL10 (IP-10) ELISA kits were from R&D Systems (Minneapolis, MN). Phorbol myristate acetate and the iron chelator deferiprone (DFP) were purchased from Sigma Aldrich (St. Louis, MS). Purified synthetic hepcidin-25 and LL-37 peptides were a kind gift from Dr. Jan Pohl (CDC, Atlanta, GA).

### Bacterial cultures

*N. gonorrhoeae* strain FA19 (GC-FA19) was grown as piliated, opacity-negative colony variants on GCB agar containing defined Supplements I and II under 3.8% (v/v) CO_2_ at 37°C as described by Shafer et al. [20]. Broth cultures of gonococci were grown in GCB broth with supplements and 0.043% (w/v) sodium bicarbonate at 37°C in a shaking water bath. Viability of gonococcal cultures was determined using dilution plating onto GCB agar and colony forming units were enumerated after 24–48 hr of incubation at 37°C in a CO_2_ incubator. Gonococci grown on GCB agar plates were resuspended in unsupplemented GCB broth and harvested by centrifugation at 5,000×*g* for 10 minutes. The bacterial pellet was washed twice with PBS and resuspended in 10 ml of tissue culture medium D-MEM without antibiotics to prepare a live bacterial inoculum for the macrophage infection assay (see below). An aliquot of bacteria was used to prepare heat-killed bacteria by heating for 10 min at 95°C prior to use in the macrophage infection assay.

### Cell cultures

THP-1 and MM6 human macrophage-like monocytic cells were obtained from the American Type Culture Collection (ATCC, Manassas, VA) and grown in RPMI1640 containing L-glutamate and supplemented with 10% FBS (v/v), 50 IU/ml of penicillin, 50 µg/ml of streptomycin, 1% sodium pyruvate (w/v) and 1% non-essential amino acids (v/v). Culture flasks were incubated at 37°C with humidity and 5% CO_2_. Murine macrophages (RAW264 from ATCC) were grown in D-MEM supplemented and incubated as noted above.

### Macrophage infection assay

Freshly grown human THP-1 and MM6 monocytic cells as well as peripheral human monocytes were harvested and adjusted to one million cells/ml without antibiotics, transferred into 6-well tissue culture plates (3 ml/well), and infected with live or heat-killed GC-FA19 at a multiplicity of infection (MOI) of 10. Cells were then incubated at 37°C with 5% CO_2_ for 5 hr or overnight. Uninfected cells in triplicate wells were also incubated simultaneously and used as a no-infection control. In some assays, monocytes were treated with the iron chelator deferiprone (DFP was added at 300 µM final concentration simultaneously upon infection with GC-FA19). Supernatants were harvested and saved at −20°C for determination of cytokine release and cells were used for RNA extraction.

### Monocyte and macrophage bactericidal assay

To determine whether gonococci survive phagocytic killing by monocytes and macrophages, we employed THP-1 macrophage-like monocytic cells and RAW264 murine macrophages. Overnight plates of GC-FA19 (Pil^+^/Opa^−^) were adjusted to an OD_600_ of 1.0 (∼1×10^8^ CFU/ml) in antibiotic-free RPMI1640 medium containing 10% heat-inactivated fetal bovine serum (FBS). To deactivate complement, FBS was heat-inactivated by incubation at 56°C for 30 min prior to use. Monocytes were freshly grown, washed, and adjusted to 1 million cells/ml in antibiotic-free RPMI1640 medium containing 10% heat-inactivated FBS as described above and were infected with GC-FA19 at an MOI of 50. Since THP-1 cells are not adherent, the infection process was initiated in 50 ml conical tubes containing 10 ml of THP-1 cell suspension. To facilitate phagocytosis of non-opsonized gonococci, the mixture of infected monocytes was centrifuged at 1300 rpm for 4 min, followed by a 10 min incubation at 37°C with 5% CO_2_ and simple agitation every 5 min. The mixture was then fully resuspended using a 10 ml pipette and further incubated for another 50 min at 37°C with 5% CO_2_ and simple agitation every 10 min. The cell mixture was then centrifuged at 1300 rpm for 4 min and washed three times with antibiotic-free medium containing 10% heat-inactivated FBS to remove extracellular gonococci. The viability of extracellular (nonphagocytosed) gonococci was assessed in the supernatants of infected cells after 1 hr of infection using the agar plate dilution method as described [21], [22]. Infected monocytes were then resuspended in the original volume using fresh antibiotic-free medium containing 10% heat-inactivated FBS. Cell suspensions were then transferred into 12-well tissue culture plates (1 ml per well) and further incubated at 37°C for 2 and 5 hr. To quantify viable adherent and intracellular gonococci at 1, 2, and 5 hr post phagocytosis, a 1 ml aliquot of the cell suspension was pelleted by centrifugation at 4000 rpm for 4 min, washed once with antibiotic-free RPMI1640 medium, and harvested by another centrifugation. The cell pellet was thoroughly resuspended in 0.01% (v/v) Triton X-100 in sterile PBS and incubated for 5 min at 37°C, then vortexed thoroughly to permeate monocyte membranes (which facilitates the retrieval of viable intracellular gonococci). The lysed cell mixture was serially diluted in sterile PBS and cultured on GCB agar plates followed by 24–48 hr of incubation at 37°C with 5% CO_2_, after which viable GC colonies were counted. In some experiments, the monocyte bactericidal assay was performed using the murine RAW264 macrophages. Since these RAW264 macrophages are adherent, cells were seeded in a 12-well tissue culture plate (1 million cells/well) and allowed to adhere overnight prior to infection with GC-FA19 at an MOI of 50 as described above. After 1 hour of the phagocytosis assay at 37°C, adherent RAW264 cells were washed three times with antibiotic-free medium containing 10% heat-inactivated FBS to remove extracellular gonococci and all fluids were carefully removed without disturbing the adherent macrophages. One ml of fresh antibiotic-free medium containing 10% heat-inactivated FBS was added to each well and infected cells were further incubated for 2 and 5 hr. Viability of adherent and intracellular gonococci was assessed by serial plating of lysed macrophages in 0.01% (v/v) Triton X-100 in PBS as described above.

### Staining of extracellular and intracellular gonococci

Viable and dead intracellular and extracellular gonococci were visualized using the LIVE/DEAD® BacLight™ Bacterial Viability Kit (Invitrogen) as described by Criss et al. [22]. Briefly, freshly grown murine RAW264 macrophages were adjusted to 1 million cells/ml and seeded on pre-cleaned glass cover slips (24×24 #1 from Surgipath, Medical Industries INC.) placed in 8-well tissue culture plates and incubated overnight as above. Adherent macrophages were washed twice with and placed in antibiotic-free medium containing 10% heat-inactivated FBS prior to infection with GC-FA19 at an MOI of 50 followed by incubation at 37°C with 5% CO_2_ for 1 hr and 5 hr. At the indicated time points, macrophages were washed three times with 0.1 M MOPS, 1 mM MgCl_2_ (MOPS/MgCl_2_) buffer, pH 7.2 to remove nonphagocytosed gonococci. Infected macrophages were then stained with BacLight™ stain components (60 µM of propidium iodide [PI] and 2.5 µM of SYTO 9) prepared in MOPS/MgCl_2_ containing 0.1% saponin to permeabilize macrophage membranes and were incubated at room temperature for 20 min in the dark with gentle rocking. Since PI is membrane impermeable, other infected macrophages in the same experiment were similarly stained with BacLight™, but without saponin, to visualize extracellular gonococci. Uninfected macrophages were also stained with BacLight™ in MOPS/MgCl_2_ with or without saponin as a control. Stained macrophages were washed twice with MOPS/MgCl_2_ and glass cover slips were inverted on glass slides and sealed immediately. Adherent and intracellular viable gonococci were examined using laser scanning confocal microscopy (Olympus IX8S1F-3, Olympus Corporation, Japan) within 30 min of mounting as described [23]. Multiple fields were examined in each individual glass cover slip and images were captured and analyzed using FV10-ASW software from Olympus.

### Minimum bactericidal concentration assay

A modified version of the minimum bactericidal concentration (MBC) assay of Shafer et al [24] was employed. Briefly, mid-logarithmic phase cultures of wild type strain FA19 and an isogenic mutant containing an insertional mutation in the *lptA* gene (*lptA::spc*), which renders gonococci hypersusceptible to cationic antimicrobial peptides due to loss of phosphoethanolamine (PEA) decoration at the 4′ lipid A phosphate [25], were normalized to an OD_600_ of 0.4 and diluted 1∶100 in 0.2x strength unsupplemented GCB broth without sodium bicarbonate (pH 7.2 or pH 5.0). Ninety µl of the diluted cultures were added to sterile 96-well polypropylene microtiter wells (Costar cat# 3879) that contained 10 µl of peptide solution. Different concentrations of polymyxin B, hepcidin-25 or LL-37 were achieved by 2-fold serial dilution in antimicrobial peptide buffer [26] (0.01% acetic acid [v/v], 0.2% fatty acid- and endotoxin-free bovine serum albumin [w/v]), before addition of bacteria. In a control well, bacteria were also mixed with buffer alone to confirm that any killing was due only to peptide activity. Microtiter plates were incubated at 37°C under 3.8% (v/v) CO_2_ for 1 hr. At the conclusion of the incubation period, 5 µl aliquots from each well were spotted onto GCB agar plates to determine the MBC of the peptides. The MBC was defined as the lowest tested peptide concentration at which no viable bacteria were recovered.

### Differentiation of THP-1 macrophages

Human THP-1 monocytes are macrophage-like monocytic cells that grow in suspension and can be differentiated into an adherent macrophages using phorbol myristate acetate (PMA). Briefly, THP-1 monocytes were treated with PMA at 20 ng/million cells and incubated at 37°C with 5% CO_2_ for one week with fresh RPMI1640 medium containing 10% FBS added every three days. Once cells became adherent, all medium was removed and replaced with fresh medium, then adherent macrophages were further incubated to form a confluent monolayer. To prepare for infection, adherent THP-1 macrophages were then harvested, counted and adjusted to 1 million cells/ml, seeded on glass cover slips placed in 8-well tissue culture plates, and allowed to adhere to glass cover slips overnight. The staining procedure was performed as described for RAW264 macrophages as mentioned above.

### Isolation of peripheral monocytes

This study was deemed exempt from Institutional Review Board (IRB) at Emory University since peripheral monocytes were completely de-identified without any link to donors' identification. However, whole blood (15 ml with EDTA) was collected from healthy donors after obtaining written informed consent under Emory University IRB approval to collect healthy donors' plasma for other unrelated studies. Monocytes used in this study were obtained from the discarded and de-identified whole blood samples leftover after the removal of plasma fraction. Peripheral monocytes were isolated using Ficoll-density gradient centrifugation (Histopaque 1077, Sigma-Aldrich Co). Isolated mononuclear cells were then cultured in RPMI1640 medium at 37°C for 2 hr to remove the non-adherent cells, followed by overnight incubation in fresh medium as described [27]. Primary peripheral monocytes were isolated from four different healthy donors and infected with live GC-FA19 at an MOI of 10 and incubated overnight. Cytokine release was measured in the supernatants using ELISA. Monocytes were harvested for RNA isolation and gene expression was measured by quantitative RT-PCR.

### Mammalian RNA isolation, quantitative Real-Time PCR and gene expression analysis

RNA was isolated using RNeasy Mini kits (Qiagen, Hilden, Germany) following the manufacturer's instructions. Briefly, infected and uninfected cells were harvested in RLT buffer containing 1% β-mercaptoethanol, passed over QiaShredder columns, and the resulting lysate was mixed in 70% ethanol and passed over RNeasy columns. Columns were washed then treated with 10 µl of RNase-free DNase (Qiagen) for 15 min at room temperature prior to RNA extraction, followed by additional washing and centrifugation. RNA was eluted with RNase-free water in 50 µl then was reverse transcribed to cDNA using the QuantiTect® Reverse Transcription kit (Qiagen) following the manufacturer's instructions. In brief, genomic DNA was eliminated using gDNA Wipeout on 1 µg of total RNA, which was then reverse transcribed in a 20 µl total volume containing reverse-transcription master mix. For reverse transcription, RNA mixture samples were incubated for 15 min at 42°C, and then 3 min at 95°C to inactivate the reverse transcriptase enzyme. The generated cDNA was diluted 1∶10 in nuclease-free molecular grade sterile H_2_O and stored at −20°C until further use. Relative gene expression was determined by quantitative RT-PCR using SYBR Green master mix (Promega, Madison, WI) and cDNA from infected and uninfected cells (as template) following the manufacturer's instructions. Gene expression fold change was calculated in reference to uninfected controls using the ΔΔC_T_ method [28]. Results were normalized to those from uninfected cells, which were used as controls for basal gene expression levels.

The following primers were used for qRT-PCR reactions: human hepcidin 5′-GACCAGTGGCTCTGTTTTCC-3′ and 5′-CACATCCCACACTTTGATCG-3′; human BDH2 5′-CAGCGTCAAAGGAGTTGTGA-3′ and 5′-TGGCGTATCAACTGTTCCTG-3′; human MMP9 5′-CTGAAATGACGTCCCTAAGT-3′ and 5′-AGGAGGTCTCACTATCTGGAT-3′; human NGAL 5′-ATGACATGAACCTGCTCGATA-3′ and 5′-TCATAGTCGTTCATTATCTTC-3′; human NRAMP1 5′-GCGAGGTCTGCCATCTCTAC-3′ and 5′-GTGTCCACGATGGTGATGAG-3′; human β-actin 5′-TCTTCCAGCCTTCCTTCCT-3′ and 5′-AGCACTGTGTTGGCGTACAG-3′; murine HAMP1 5′-AGAAAGCAGGGCAGACATTG-3′ and 5′-CACTGGGAATTGTTACAGCATT-3′; murine LCN2 5′-TGCCACTCCATCTTTCCTGTT-3′ and 5′-GGGAGTGCTGGCCAAATAAG-3′; murine BDH2 5′-GAGAACAGATGTGTGTACAGTGCTACC-3′ and 5′-CTAGGAAGGGCCTGTCTTCCCAGC-3′; murine NRAMP1 5′-GACACAGCAGAGCAATTGGA-3′ and 5′-GGGAACTGGAGTCACCTTCA-3′; murine GAPDH 5′-ACCTGCCAAGTATGATGACATCA-3′ and 5′-GGTCCTCAGTGTAGCCCAAGAT-3′. Ferroportin QuantiTect primers (Hs_SLC40A1_1_SG) were purchased from Qiagen.

### Bacterial RNA isolation and quantitative RT-PCR

Expression of iron-responsive genes in intracellular GC-FA19 living in monocytes was assessed by quantitative RT-PCR. Briefly, THP-1 cells were infected with GC-FA19 as mentioned above (see monocyte and macrophage bactericidal assay section) and GC-FA19 was harvested for RNA extraction. Intracellular GC-FA19 (phagocytosed gonococci) were harvested by lysing infected THP-1 cells using the Qiagen Shredder as mentioned above. Bacterial RNA was isolated using RNeasy columns (Qiagen) and genomic DNA was removed by DNase treatment and gDNA Wipeout (Qiagen) following the manufacturer's instructions. The absence of contaminating genomic DNA was confirmed by using purified RNA as template in a PCR reaction and visualization by gel electrophoresis. The extracted bacterial RNA was then reverse transcribed to cDNA using the QuantiTect Reverse Transcriptase kit (Qiagen) following the manufacturer's instructions. Quantitative RT-PCR was performed using the generated cDNA (undiluted) and results were normalized to 16S rRNA expression for each individual sample. The expression of iron-responsive genes *fetA*, *tbpA*, *tbpB*, *mpeR*, and non-iron-responsive genes *rmpM* and *serC* in monocyte-associated GC-FA19 after 5 hr of infection was compared to that after 1 hr of infection using the ΔΔC_T_ method [28]. GC-FA19 specific primers used in this study: *tbpA* forward: 5′-TTTCGACACGCGCGATATGA-3′, reverse: 5′-AGTCCGCCGTATTTGTGGTT-3′; *tbpB* forward: 5′-AGGGCAAGGCGACAAATACA-3′, and reverse: 5′-CGAATCAGTTTGCCCGTCAA-3′; *fetA* forward: 5′-AGAGTTTGCCGTCAGCGAAA-3′, and reverse: 5′-TAGGCGTTGGCATCCAGTTT-3′; *mpeR* forward: 5′-AAACAGCCCGGTTTGCATCT-3′, and reverse: 5′-GCGCAGTTGTGGCTGAAATT-3′; *rmpM* forward: 5′-AAGCCAAGGTCGCGTAGAAT-3′, and reverse: 5′-GGCGCGCAATGAATCCTTAT-3′; *serC* forward: 5′-TGTTGCCTGAAGCTGTGTTG-3′, and reverse: 5′-TGTTCCGCATGATGCAGGAT-3′; *16S* forward: 5′-CCATCGGTATTCCTCCACATCTCT-3′, and reverse: 5′-CGTAGGGTGCGAGCGTTAATC-3′.

### Cytokine release quantification

The cytokines TNFα, IL-1β, IL-6, MCP-1, CXCL10 (IP-10), and NGAL released from THP-1 cells or from peripheral human monocytes were quantified by DuoSet ELISA (R&D Systems, Minneapolis, MN) as previously described [29], [30].

### Nitric oxide induction by murine macrophages

Freshly grown adherent RAW264 macrophages were harvested and adjusted to one million cells/ml without antibiotics, then transferred into 6-well tissue culture plates (3 ml/well) and infected with live GC-FA19 at an MOI of 10 and incubated at 37°C with 5% CO_2_ overnight. Nitric oxide release was quantified in the supernatants using the Greiss chemical method as previously described [30].

### Cytospin and cellular staining

To monitor intracellular gonococci residing in macrophages, 100 µl of infected and uninfected THP-1 and MM6 cells were subjected to cytospin centrifugation for 5 min at 500 rpm using Cytospin 4 (Thermo Scientific). Cells deposited on glass slides were allowed to dry for at least 5 min prior to staining with the modified Wright-Giemsa stain Diff-quick® (Dade Behring, Newark, DE, USA) following the manufacturer's instructions. Cellular morphology was examined by light microscopy and digital images of 10 different fields per sample were saved.

### Measurement of iron retention in infected monocytic cells

Intracellular labile iron reflecting iron retention in infected and uninfected THP-1 and MM6 cells was determined using the well-established Calcein-AM method [31]. Infected and uninfected cells were washed and placed in RPMI1640 medium supplemented with 10% FBS, and 0.5 µM Calcein-AM was added to 1 million cells/ml at 37°C and incubated for 15 min. Calcein-AM-loaded cells were washed twice with PBS to remove extracellular Calcein-AM. Cells were resuspended in HBSS at 2 million cells/ml and 100 µl aliquots were transferred into quadruplicate wells in black 96-well plates. After 20 min of incubation, fluorescence was determined at 485 nm (excitation) and 535 nm (emission) wavelengths using a Bio-Tek Synergy 2 Instrument as described [31].

### Western immunoblotting analysis

THP-1 cells were infected with GC-FA19 as above or treated with 1 ng/ml of exogenous hepcidin protein (RDG International Inc., USA) and incubated for 5 hrs. Untreated cells were used as a control. Treated and untreated cells were lysed with RIPA buffer containing protease inhibitors and 20 µg of cell protein extracts were loaded into a Tris-glycine 4–20% SDS polyacrylamide gel (Novex, Invitrogen, Carlsbad, CA) and subjected to electrophoresis. Resolved proteins were transferred onto a nitrocellulose membrane and blocked with 3% BSA overnight. Hepcidin immunoreactive bands were detected with the primary anti-hepcidin polyclonal antibody diluted 1∶1000 (rabbit anti-human hepcidin polyclonal antibody from Ray Biotech Inc., Norcross, GA). The membranes were washed three times with TBS-T buffer, incubated with the goat anti-rabbit secondary antibody alkaline phosphatase conjugate (diluted 1∶1000), washed an additional three times then developed using SigmaFast BCIP/NBT (Sigma Aldrich).

### Statistical analysis

Mean values ± SD and *P* values (Student *t* test) of at least three independent determinations were calculated with Microsoft Excel software.

## Results

### *N. gonorrhoeae* survives in association with monocytes and macrophages and responds to an iron-limited environment

**Figure 1:**
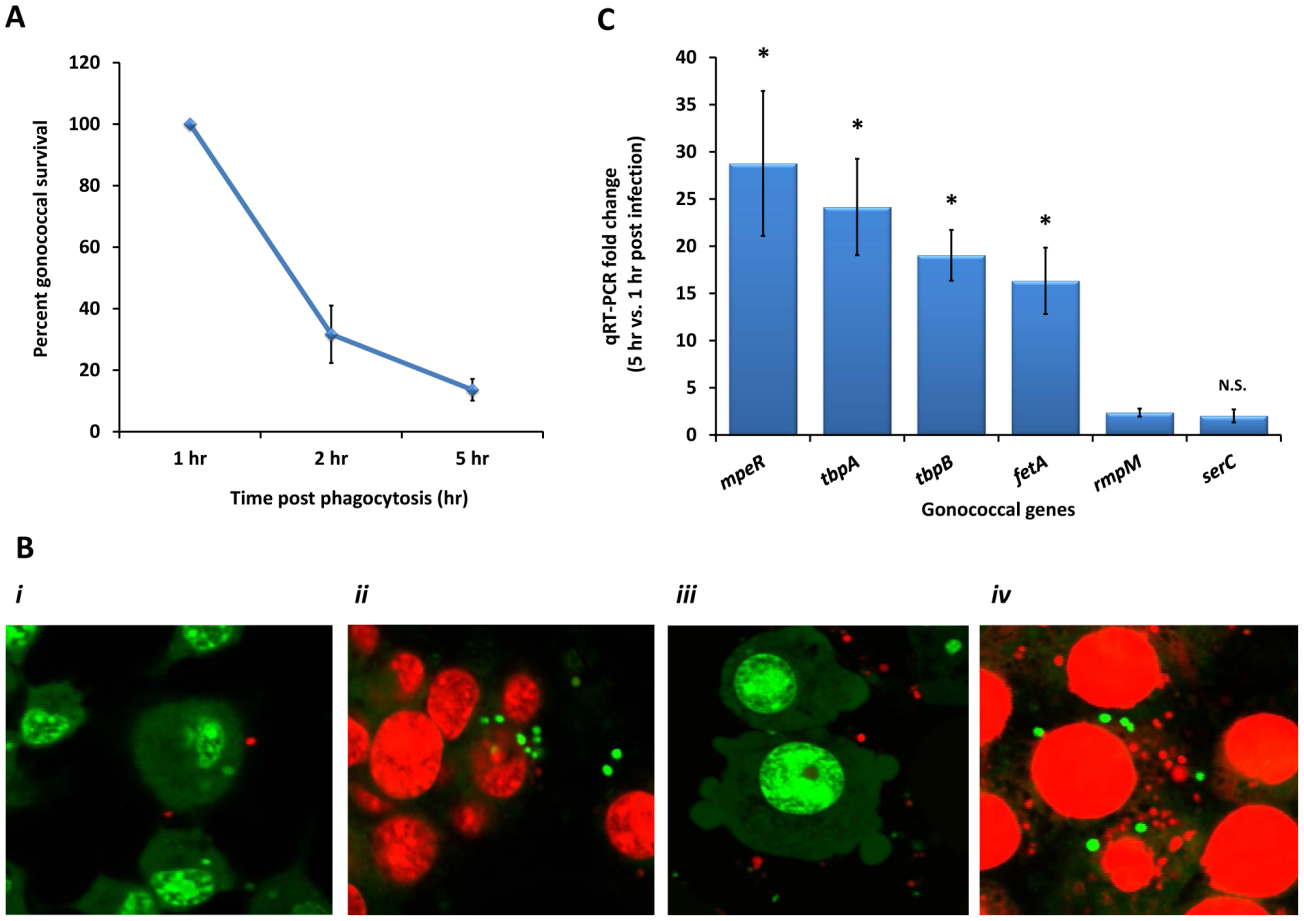
*N. gonorrhoeae* survives in association with monocytes. **A.** Survival of gonococci with human THP-1 monocytes at 2 and 5 hr post infection. Percent monocyte-associated gonococcal survival is calculated in reference to the number of viable gonococci cultured from infected THP-1 cells after 1 hr of phagocytosis. Error bars are ± standard deviation from the mean of 4 independent experiments. **B**: Viability of adherent and intracellular and extracellular GC-FA19 in differentiated THP-1 cells, i.e. adherent macrophages, were visualized with BacLight staining where red is dead and green is live gonococci. Adherent THP-1 cell membranes were permeabilized with 0.1% saponin to stain phagocytosed gonococci. ***i***: 1 hr post infection stained without saponin. ***ii***: 1 hr post infection stained in the presence of 0.1% saponin. ***iii***: 5 hr post infection stained without saponin. ***iv***: 5 hr post infection stained in the presence of 0.1% saponin. These data are representative of two independent experiments. **C**: Quantitative RT-PCR of iron-responsive and -unresponsive genes in monocyte-associated GC-FA19 at 5 hrs post infection compared to 1 hr post infection. Error bars represent SD from the mean of three independent experiments. * *P* values (<0.005) were calculated in comparison to *rmpM* gene expression. N.S.: not significant.

The strict human pathogen *N. gonorrhoeae* evades host innate defenses and survives in neutrophils [5], [23], macrophages [32] and epithelial cells [33]. Macrophages play a central role in limiting iron bioavailability during infection to prevent bacterial growth [7]. Therefore, we examined whether gonococci can cope with the iron-limiting conditions within monocytes/macrophages and survive intracellularly. We determined the viability of gonococci during infection using a macrophage bactericidal assay adapted from previously described methods [22], [32]. THP-1 monocytes were infected with unopsonized GC-FA19 at an MOI of 50 and the viability of extracellular and monocyte-associated gonococci was determined at 1, 2, and 5 hr post infection. We found that *N. gonorrhoeae* survived killing by human THP-1 monocytes at 1, 2, and 5 hr post infection (Figure 1A). In this respect, although 50% of the total gonococcal inoculum was killed within 1 hr of exposure to monocytes, 47% of this inoculum was viable extracellularly while approximately 3% was phagocytosed (data not presented). During the subsequent four hours, phagocytosed gonococci could be retrieved viable. Of the gonococci that were adherent or intracellular and viable at 1 hr, approximately 30% remained viable at the 2 hr time point, while at least 15% remained viable to the end (5 hr) of the phagocytic killing period (Figure 1A). The viability of gonococci (adherent and intracellular) in differentiated THP-1 macrophages (Figure 1B) was visualized using bacterial live/dead staining as described [22]. Further, gonococcal survival was also observed when murine RAW264 macrophages were infected with unopsonized GC-FA19 (see below). These data indicate that adherent and intracellular *N. gonorrhoeae* can survive phagocytic killing by monocytes and macrophages.

In order to determine the transcriptional response of gonococci to the reported iron-limiting conditions inside of phagocytic cells [7], [12], we investigated the expression of gonococcal iron-responsive genes that encode the transferrin-binding protein complex (*tbpA* and *tbpB*), an enterobactin-like siderophore receptor (*fetA*) and a transcriptional activator (*mpeR*) of *fetA* that is negatively regulated by the gonococcal ferric uptake regulator (Fur) in the presence of iron; two non-iron-responsive genes that encode the gonococcal reduction modification protein (*rmpM*) and phosphoserine aminotransferase (*serC*) were also tested as controls [34], [35], [36], [37]. We found that these iron-responsive genes were significantly upregulated in monocyte-associated gonococci at 5 hr compared with 1 hr of phagocytosis (Figure 1C). In contrast, the gene expression of *rmpM* and *serC* was largely unchanged. Taken together, the data suggest that *N. gonorrhoeae* survives in association with monocytes and macrophages and responds to iron limitation by upregulating iron-responsive genes to facilitate iron acquisition.

### Innate immune recognition of *N. gonorrhoeae* in infected monocytes

**Figure 2:**
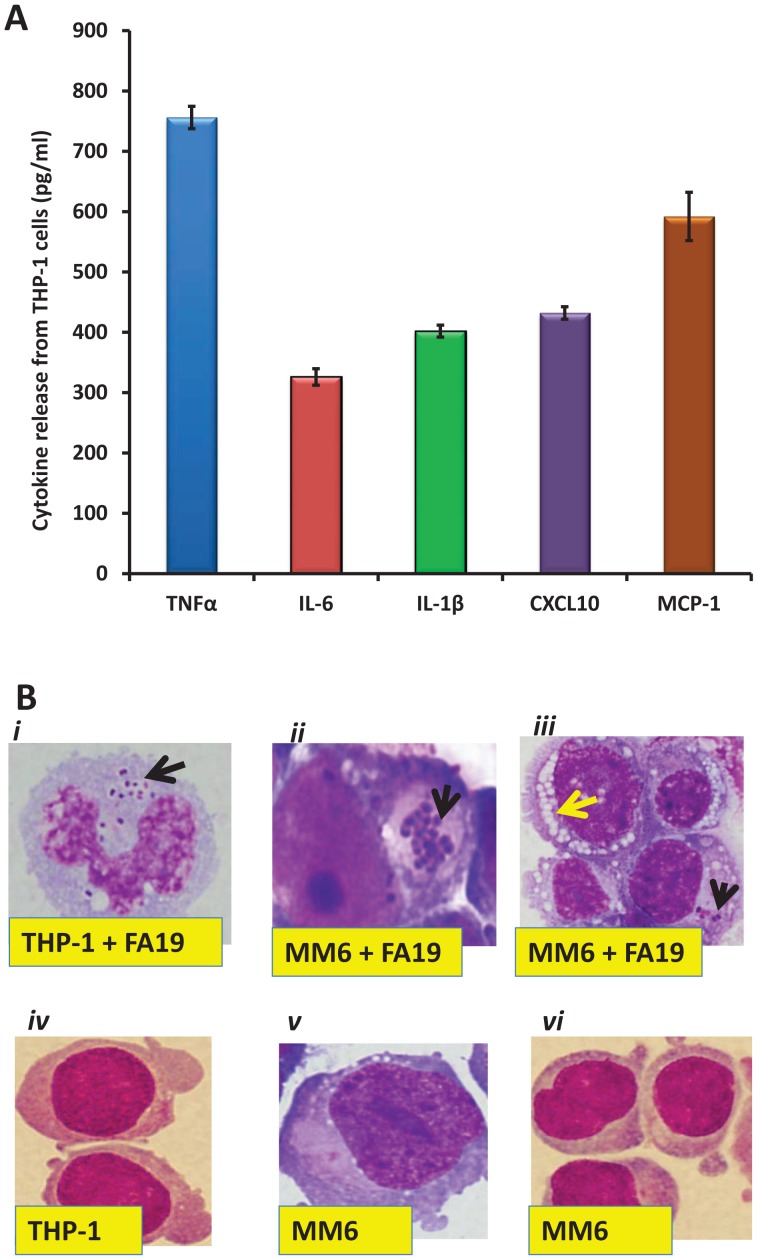
Cytokine release from THP-1 cells infected with *N. gonorrhoeae* strain FA19. **A.** Human macrophage-like monocytic THP-1 cells were infected with GC-FA19 at an MOI of 10 and incubated overnight. Released cytokines TNFα, IL-6, IL-1β and chemokines CXCL10 (IP-10) and MCP-1 were quantified in the supernatants of infected THP-1 cells using ELISA. Cytokine release was not detectable in the supernatants of uninfected THP-1 cells. Error bars represent the SD from the mean of triplicate readings and data are representative of three independent experiments. **B.** Intracellular gonococci (black arrows) were visualized by differential staining of infected THP-1 (***i***) and MM6 (***ii***) cells showing cytoplasmic vacuoles (yellow arrow) (***iii***) indicating monocyte activation and differentiation into macrophage-like cells, shown in the upper panel. Uninfected THP-1 (***iv***) and MM6 (***v, vi***) cells are shown in the lower panel.

**Figure 3:**
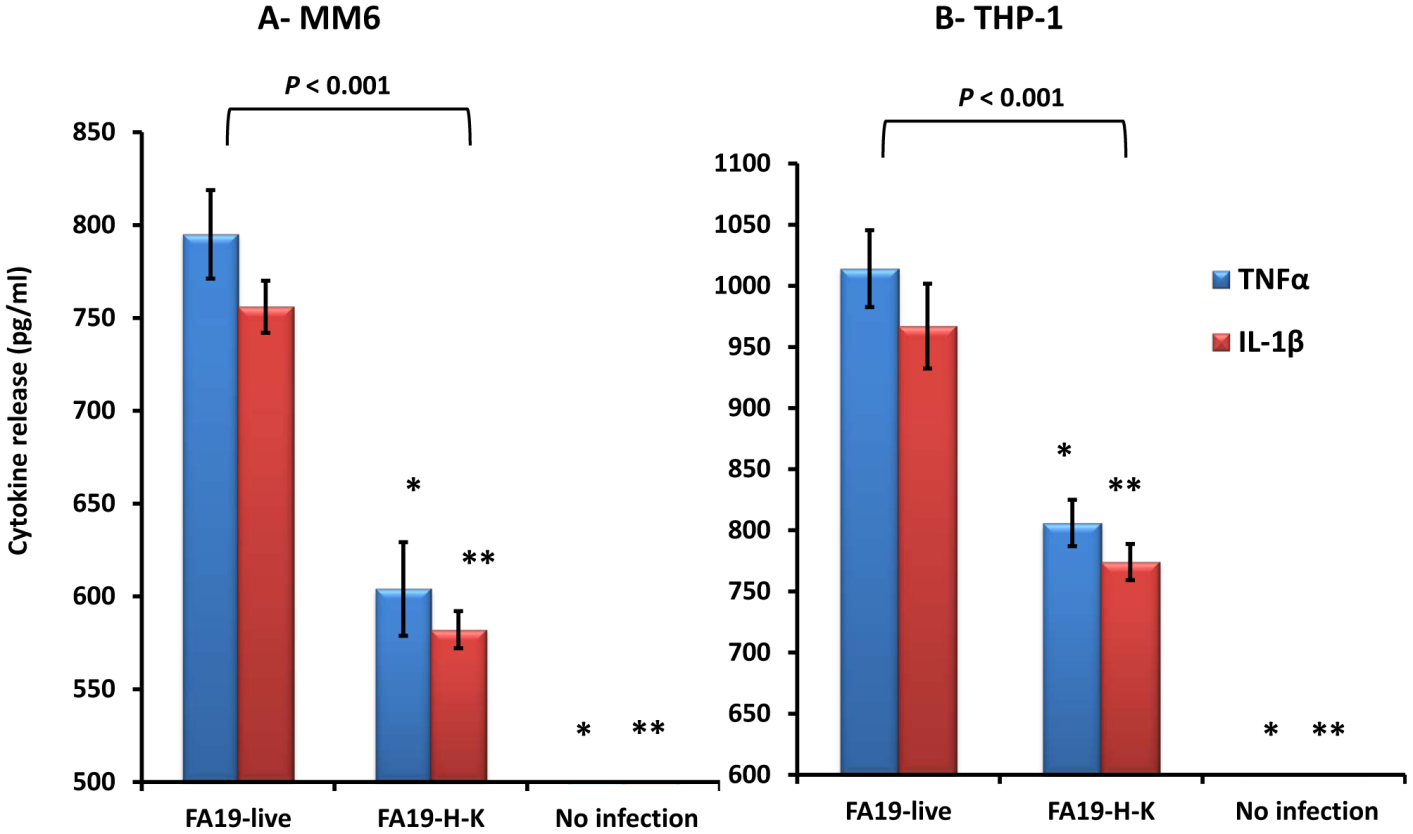
Infection with live gonococci induces more cytokine release than infection with heat-killed gonococci. Human macrophage-like monocytic MM6 (**A**) and THP-1 (**B**) cells were infected with live or heat-killed (H-K) GC-FA19 at an MOI of 10 and incubated overnight. Released cytokines TNFα and IL-1β were quantified in the supernatants of infected cells by ELISA. Cytokine release was not detectable in the supernatants of uninfected cells. Error bars represent the SD from the mean of triplicate readings and data are representative of two independent experiments. *P* values (<0.001) were calculated for values of infections with live GC-FA19 compared to heat-killed and * TNFα, ** IL-1β in reference to no infection values.

Upon recognition of invading pathogens, macrophages secrete inflammatory mediators including cytokines and chemokines required for orchestrating innate immune defenses that limit or clear infection. In order to assess if we could detect classical macrophage responses to a bacterial infection, we first examined if their infection by gonococci influenced expression of cytokines and chemokines. As expected, human THP-1 monocytes recognized the presence of *N. gonorrhoeae* strain FA19 (GC-FA19) and released cytokines TNFα, IL-6 and IL-1β [38] as well as chemokines CXCL10 (also known as IP-10) and MCP-1 (Figure 2A). The presence of intracellular gonococci and morphological changes such as the appearance of vacuoles associated with macrophage activation were visualized by microscopic examination of Wright-Giemsa stained smears of infected and uninfected THP-1 and MM6 cells (Figure 2B). These data indicated that monocytes can sense the presence of intracellular gonococci and respond accordingly. We next examined if viability of GC-FA19 influences pathogen sensing and cytokine release from monocytes. We found that infection with live GC-FA19 induced significantly more TNFα and IL-1β release from MM6 (Figure 3A) and THP-1 cells (Figure 3B) when compared to a heat-killed (H-K) preparation of GC-FA19, indicating that monocytes recognized the presence of both live and dead GC-FA19 infection, but responded more robustly to infection by live GC-FA19, possibly due to the growth or biologic activities of viable gonococci. Having established that we could detect classical macrophage responses due to infection by gonococci, we next evaluated if this infection also influenced expression of host genes involved in modulating levels of bioavailable iron.

### *N. gonorrhoeae* modulates monocyte iron-limiting innate immune defenses

**Figure 4:**
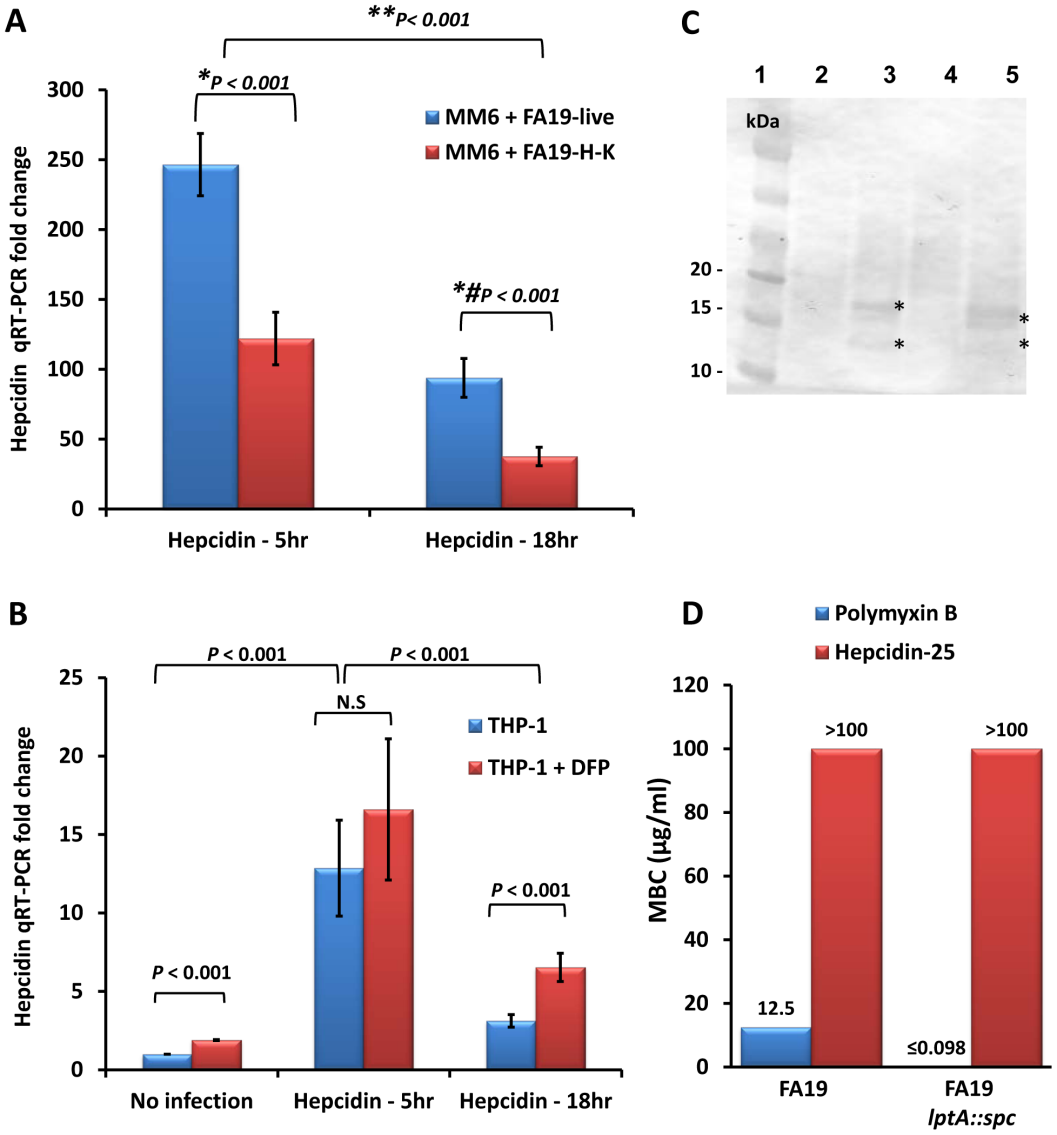
Hepcidin expression in monocytes is highly upregulated upon infection with live gonococci. **A:** Human macrophage-like monocytic MM6 cells were infected with live or heat-killed (H-K) GC-FA19 and incubated for 5 hr or 18 hr. **B:** THP-1 cells were infected with live GC-FA19 in the presence or absence of 300 µM of the iron chelator deferiprone (DFP) and incubated for 5 hr or 18 hr. RNA was extracted from infected cells and from controls (uninfected cells) and hepcidin gene expression was assessed by quantitative RT-PCR and normalized to that of β-actin. The fold change in hepcidin gene expression was calculated in reference to uninfected controls using the ΔΔCT method. Error bars represent the SD from the mean of quadruplicate wells and data are representative of three independent experiments. **P values (<0.001) were calculated for values of hepcidin expression at 5 hr or 18 hr post infection. *P and *#*P* values (<0.001) were calculated for values of hepcidin expression upon infection with live GC-FA19 compared to heat-killed (H-K) at 5 hr and 18 hr post infection, respectively. N.S.: not significant. **C:** Western blot analysis of hepcidin protein expression in THP-1 cell extracts detected with polyclonal anti-hepcidin antibody. Lane 1: molecular weight marker; Lane 2: Untreated THP-1 cells; Lane 3: THP-1 cells treated with exogenous hepcidin (1 ng/ml); Lane 4: Uninfected THP-1 cells; Lane 5: THP-1 infected with GC-FA19. *Anti-hepcidin immunoreactive bands might be dimers of prohepcidin protein (∼16–18 kDa) or processed hepcidin (∼12 kDa). **D:** Activity of synthetic hepcidin-25 antimicrobial peptide against *N. gonorrhoeae* FA19 WT and the FA19 *lptA::spc* isogenic mutant as determined by minimum bactericidal concentration (MBC) assay. The model antimicrobial peptide, polymyxin B, is active against FA19 and highly active against the FA19 *lptA::spc* mutant, and was used as a control. Data are representative of four independent experiments. For the FA19 *lptA::spc* mutant, experiments were performed twice.

During bacterial infection the host limits the bioavailability of iron by inducing hepcidin, which retains iron in macrophages and as a consequence, limits bacterial growth due to sequestration of this important nutrient. Although *N. gonorrhoeae* survives intracellularly in neutrophils, macrophages, and epithelial cells [5], [32], [33], it is not known how or if it modulates the iron-limiting innate immune defenses in macrophages. To test this possibility, we investigated hepcidin expression by quantitative RT-PCR in human monocytic cell lines MM6 and THP-1 infected with GC-FA19 for 5 or 18 hr. We found that hepcidin gene expression was highly upregulated in MM6 cells infected with live or heat-killed gonococci when assessed at 5 hr and 18 hr post infection, with the highest expression seen at 5 hr (Figure 4A), but live gonococci seemed more proficient in this capacity. This suggests that monocytes can sense and respond more robustly to infection by live *N. gonorrhoeae* and supports our results in Figure 3. Since hepcidin expression is modulated by iron levels, we next examined the effect of iron chelation on hepcidin expression in monocytes. To this end, THP-1 monocytes were treated with 300 µM of the iron chelator deferiprone (DFP) at the time of infection with live gonococci. The results show that iron chelation upregulated basal levels of hepcidin gene expression in uninfected THP-1 cells and further increased hepcidin gene expression in infected THP-1 cells at 5 and 18 hr post infection, with the highest hepcidin gene expression seen at 5 hr (Figure 4B). Hepcidin protein induction in GC-FA19 infected, but not uninfected, THP-1 monocytes was confirmed by immunoblotting with anti-hepcidin antibody (Figure 4C). Based on this observed induction of hepcidin production, we tested whether purified hepcidin-25 antimicrobial peptide might exert anti-gonococcal activity. Although it reportedly lacks strong antibacterial action against clinical strains of *Escherichia coli* and *Klebsiella pneumoniae* [39], hepcidin-25 seems to have some activity against *Mycobacterium tuberculosis* [40]. In order to learn if hepcidin-25 could kill gonococci, we compared its activity to a model cationic antimicrobial peptide (polymyxin B; PMB) that has potent anti-gonococcal activity *in vitro* [25]. For this purpose, we used wild-type gonococcal strain FA19 and an isogenic mutant, FA19 *lptA::spc* (the latter is hypersusceptible to cationic antimicrobial peptides due to loss of PEA decoration of lipid A [25], which also renders gonococci less fit *in vivo* as assessed using experimental female mouse and human male models of genital tract infection [41]). We found that in contrast to PMB, hepcidin-25 lacked anti-gonococcal activity *in vitro* (the minimum bactericidal concentration [MBC] of PMB against wild-type FA19 was 12.5 µg/ml, while that of hepcidin-25 was >100 µg/ml) even against the cationic antimicrobial peptide-hypersusceptible strain FA19 *lptA::spc* (Figure 4D). In additional contrast to hepcidin-25, we found (data not presented) that the human cathelicidin LL-37 exerted potent anti-gonococcal activity *in vitro* (MBC = 6.25 µg/ml), which is consistent with previous studies [24]. Since the antibacterial activity of hepcidin-25 has been reported to increase at acidic pH [39], [42], we also tested whether hepcidin-25 would exert anti-gonococcal activity in a pH-dependent manner. We found (data not presented) that hepcidin-25 lacked anti-gonococcal activity when tested against FA19 WT at pH 7.2 (MBC>100 µg/ml) and at pH 5.0 (MBC>100 µg/ml).

**Figure 5:**
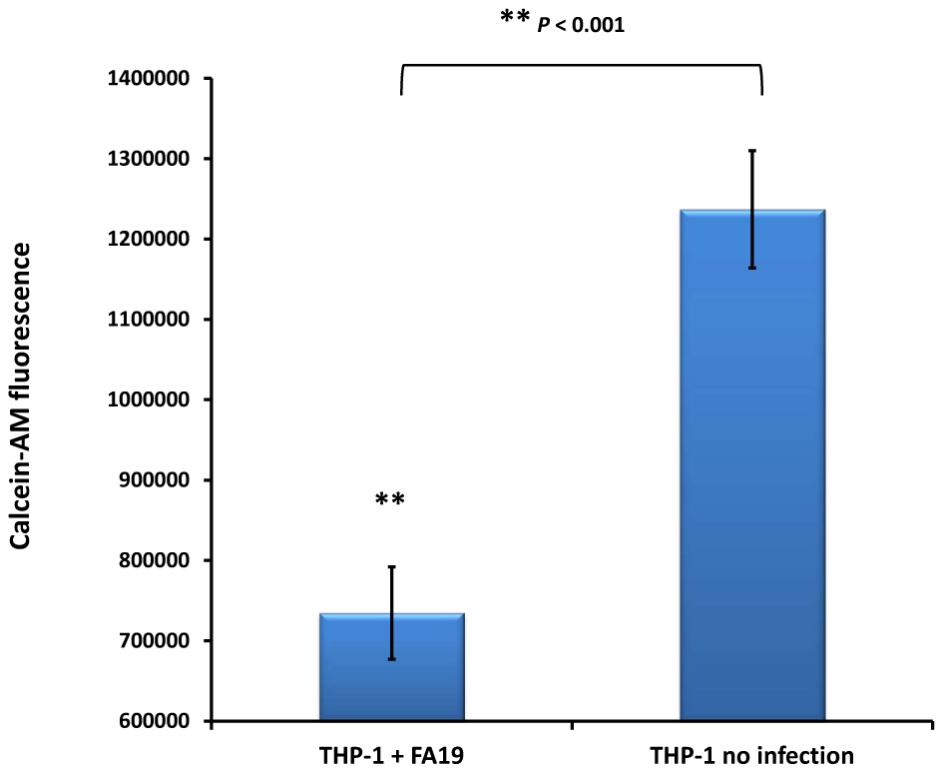
Infection with gonococci induces iron retention in monocytes. THP-1 macrophage-like monocytic cells were infected with GC-FA19 at an MOI of 10 overnight and iron retention in monocytes was determined using the Calcein-AM fluorescent probe method. Uninfected cells were incubated simultaneously and used as controls. Calcein-AM fluorescence was measured by excitation at 488 nm and emission at 528 nm wavelength (see methods). Calcein-AM fluorescence is quenched upon binding iron and is therefore inversely correlated with intracellular iron accumulation. Error bars represent the SD from the mean of quadruplicate readings and data are representative of three independent experiments. **P values (<0.001) were calculated in reference to no infection values.

**Figure 6:**
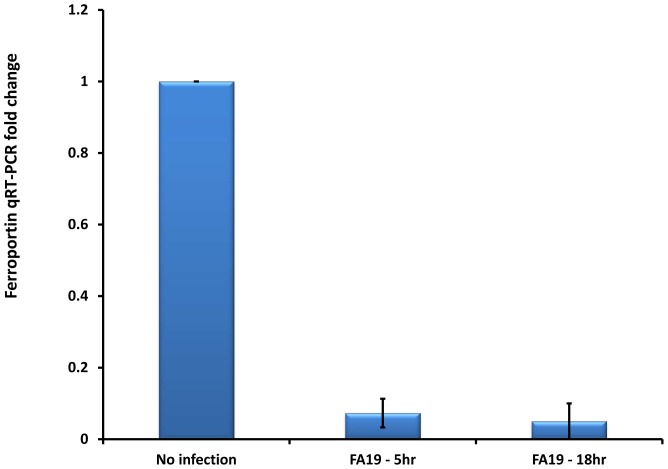
Gonococcal infection downregulates ferroportin gene expression in human THP-1 monocytes. THP-1 cells were infected at an MOI of 10 with live GC-FA19 and incubated for 5 or 18 hr. RNA was extracted from infected and uninfected cells. Ferroportin gene expression was assessed by quantitative RT-PCR and normalized to that of β-actin. Ferroportin gene expression fold change was calculated in reference to uninfected controls. Error bars represent the SD from the mean of at least quadruplicate wells and data are representative of two independent experiments.

Hepcidin induction causes iron retention in macrophages, i.e. increases the cytosolic labile iron pool (LIP), by binding ferroportin leading to its internalization and degradation thereby inhibiting iron export [6], [13]. To confirm that upregulation of hepcidin in GC-FA19 infected monocytes causes iron retention, we measured the labile iron pool in infected THP-1 monocytes (Figure 5) using the Calcein-AM fluorescent probe [31]. Upon binding labile (free) iron, Calcein-AM fluorescence is quenched and is therefore inversely correlated with labile intracellular iron accumulation. We found that Calcein-AM fluorescence was significantly quenched in THP-1 monocytes infected with GC-FA19 when compared to uninfected monocytes (Figure 5). Similar results were seen when MM6 monocytes were infected with gonococci (data not shown), suggesting that the LIP is increased upon infection with gonococci. To further confirm that hepcidin upregulation caused iron retention in infected monocytes, we investigated the expression of the only known iron exporter, ferroportin (also known as SLC40A1). We found that ferroportin gene expression was significantly downregulated in THP-1 monocytes infected with gonococci (Figure 6). Taken together, these data suggest that *N. gonorrhoeae* infection of monocytes, especially with live gonococci, upregulates hepcidin and downregulates ferroportin to retain iron in monocytes, which would increase the LIP and facilitate intracellular iron acquisition.

**Figure 7:**
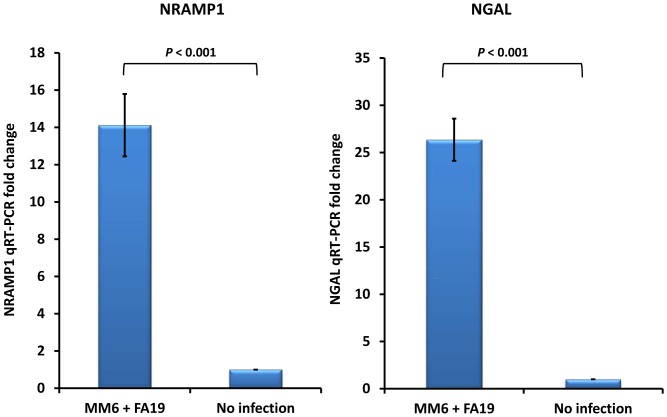
Gonococcal infection upregulates expression of the cytosolic iron transporter NRAMP1 gene and the antibacterial iron carrier NGAL gene in monocytes. Human macrophage-like monocytic MM6 cells were infected with live GC-FA19 at an MOI of 10 and incubated for 18 hr. RNA was extracted from infected cells and from controls (uninfected cells). NRAMP1 and NGAL gene expression was assessed by quantitative RT-PCR and normalized to that of β-actin. NRAMP1 and NGAL gene expression fold change was calculated in reference to uninfected controls. Error bars represent the SD from the mean of at least quadruplicate wells and data are representative of three independent experiments. *P* values (<0.001) were calculated in reference to no infection values.

Since cellular iron homeostasis is also tightly controlled by NRAMP1 and NGAL, we evaluated if their expression is influenced by gonococcal infection. NRAMP1, the intracellular iron transporter, is important in cellular iron recycling since it transports iron from the late endosome and phagolysosome. Herein, iron-containing proteins such as ferritin and transferrin are degraded and recycled to the cytosol, which increases labile cytosolic iron. NGAL is an iron carrier protein that shuttles and delivers liganded iron needed for cellular growth and differentiation. NGAL has been shown to shuttle the mammalian siderophore 2,5-DHBA [17] and also plays an important role in iron-limiting innate immune defenses since it exerts antibacterial function by sequestering bacterial siderophores, hence limiting bacterial growth [9]. We investigated NRAMP1 expression by quantitative RT-PCR in human macrophage-like monocytic cell lines MM6 and THP-1 infected with GC-FA19 for 18 hr. We found that NRAMP1 gene expression was highly upregulated in human MM6 (Figure 7) and THP-1 (data not shown) monocytes infected with GC-FA19. Similarly, we found that expression of NGAL was highly upregulated in MM6 (Figure 7) and THP-1 (data not shown) cells upon infection with GC-FA19.

### *N. gonorrhoeae* downregulates the gene encoding the labile iron-detoxifying enzyme BDH2

**Figure 8:**
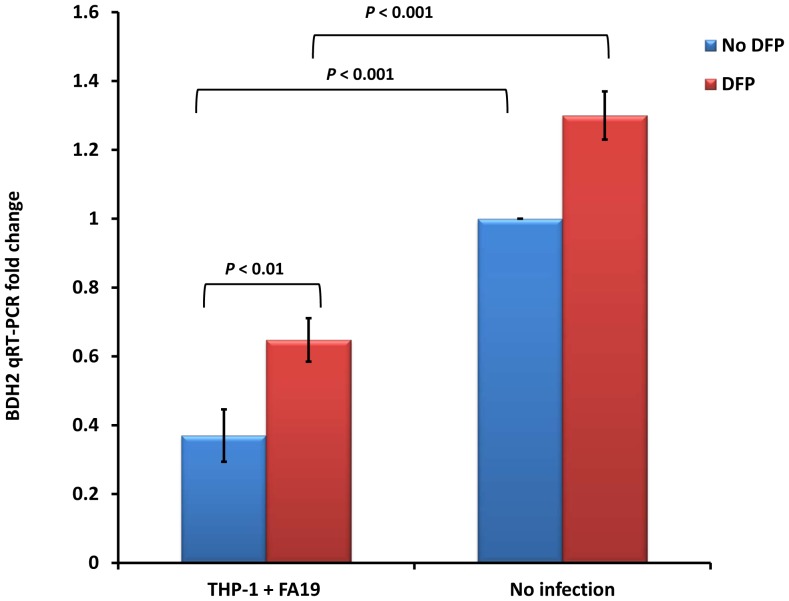
Gonococcal infection downregulates expression of the labile iron-detoxifying enzyme BDH2-encoding gene in monocytes. Human macrophage-like monocytic THP-1 cells were infected with GC-FA19 at an MOI of 10 in the presence or absence of 300 µM of deferiprone (DFP) (an iron chelator) and incubated for 18 hr. RNA was extracted from infected cells and from controls (uninfected cells) and BDH2 gene expression was assessed by quantitative RT-PCR and normalized to that of β-actin. BDH2 gene expression fold change was calculated in reference to uninfected controls. Error bars represent the SD from the mean of at least quadruplicate wells and data are representative of three independent experiments. *P* values (<0.01) were calculated in reference to no infection values.

To maintain homeostatic iron regulation, the host detoxifies free iron by multiple mechanisms [10]. Thus, iron retention increases the LIP which in turn increases the toxicity of ROS generation through the iron-dependent Fenton reaction [43]. Recently, the enzyme termed BDH2 was discovered to play a crucial role in intracellular iron homeostasis since its transcript possesses an iron responsive element (IRE) [16], [17]. BDH2 is an EntA homologue and EntA is the protein responsible for producing the bacterial siderophore 2,3-dihydroxybenzoic acid (2,3-DHBA) known as enterobactin [44]. Similarly, BDH2 was discovered to mediate the synthesis of the mammalian siderophore 2,5-DHBA that binds free iron [17]. BDH2-depleted cells were found to accumulate free iron and have increased oxidative stress [17]; therefore, BDH2 plays a very important role in detoxifying labile iron and maintaining intracellular iron homeostasis. We hypothesized that *N. gonorrhoeae* infection of monocytes would downregulate BDH2 expression to sustain the LIP and thus facilitate iron acquisition. We found that gonococcal infection downregulated BDH2 gene expression in human THP-1 monocytes as assessed by qRT-PCR (Figure 8). Since iron chelation induces BDH2 expression [16], we treated THP-1 monocytes with 300 µM of DFP at the time of infection with GC-FA19. We found that even with iron chelation, BDH2 gene expression was still downregulated by *N. gonorrhoeae* infection (Figure 8). These data suggest that gonococcal infection of monocytes likely results in reduced synthesis of the mammalian siderophore 2,5-DHBA. Thus, downregulation of the BDH2 gene would increase the labile iron pool and, consequently, facilitate intracellular iron acquisition by gonococci. To our knowledge, this is the first report to demonstrate that bacterial infection of a host cell can decrease BDH2 gene expression.

### *N. gonorrhoeae* modulates iron-limiting innate immune defenses in primary peripheral human monocytes

**Figure 9:**
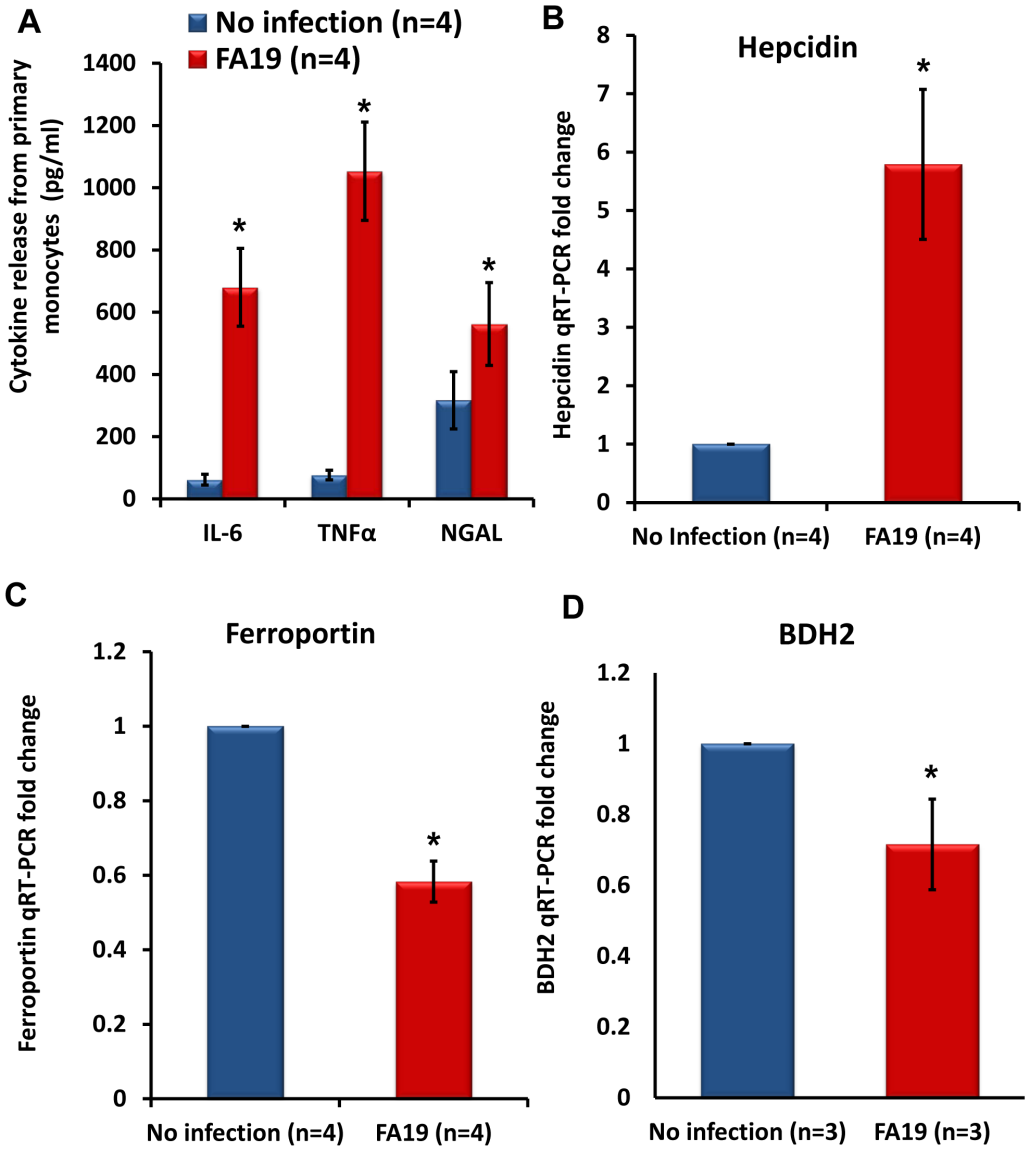
Gonococcal infection of peripheral monocytes from healthy human donors induces cytokine release, upregulates hepcidin and downregulates ferroportin and BDH2 gene expression. A: Peripheral monocytes derived from healthy donors were infected with GC-FA19 at an MOI of 10 and incubated overnight. Released cytokines IL-6 and TNFα and the antibacterial protein NGAL were quantified in the supernatants of infected monocytes by ELISA. Error bars represent the SD from the mean of four different healthy donors, each assayed in duplicate readings. B, C and D: Hepcidin, ferroportin, and BDH2 gene expression in healthy donor monocytes infected with GC-FA19 used in panel A above was determined by quantitative RT-PCR and normalized to uninfected monocytes. Error bars represent the SD from the mean fold change in gene expression from different healthy donors, each assayed in triplicate. *P value <0.01.

**Figure 10:**
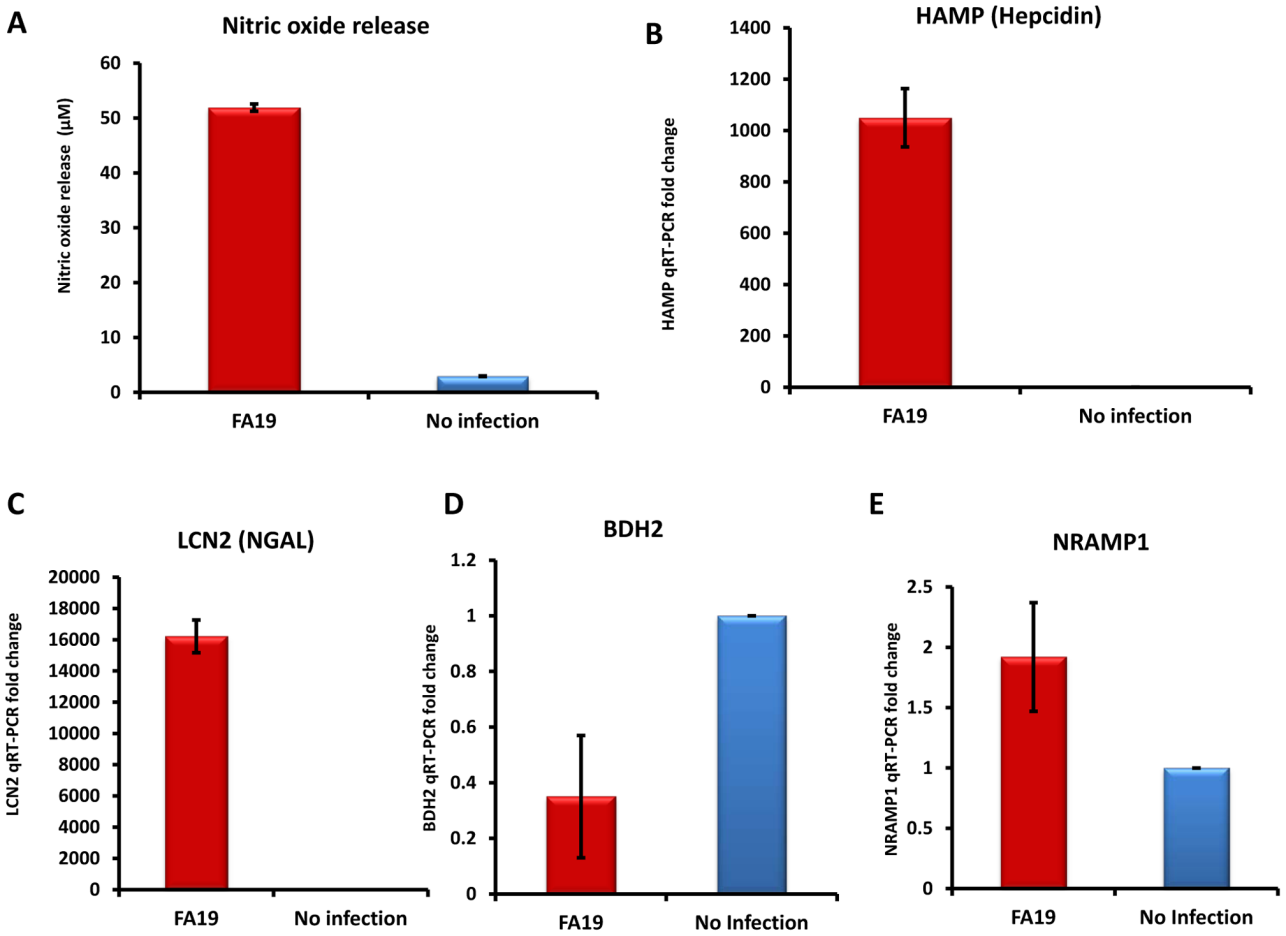
Gonococcal infection modulates iron-limiting innate immune defenses in murine RAW264 macrophages. **A**: Nitric oxide release from RAW264 cells infected with GC-FA19 was measured after 18 hr of incubation. Iron-regulated gene expression was determined by qRT-PCR from RNA extracted from the RAW264 infected cells used in the experiment shown in panel **A**: Hepcidin (**B**); LCN2 (**C**); BDH2 (**D**); NRAMP1 (**E**). Error bars represent the SD from the mean fold change of qRT-PCR, each assayed in triplicate. This result is representative of two independent experiments.

**Figure 11:**
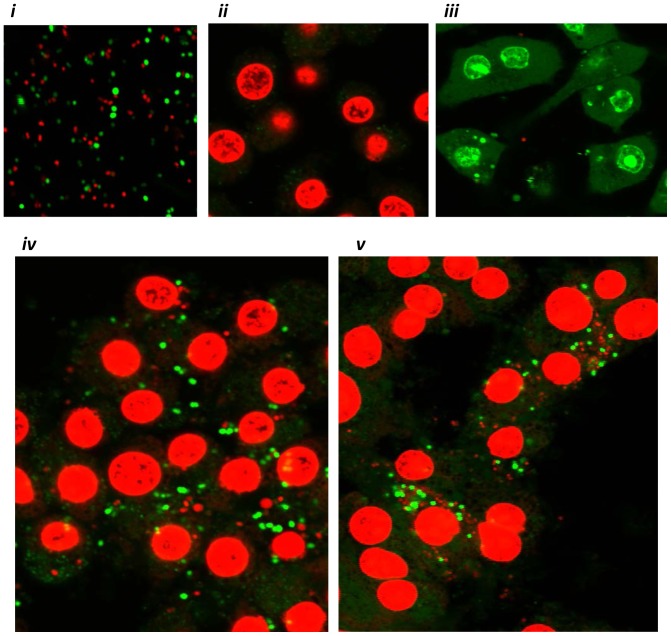
*N. gonorrhoeae* survives in association with murine RAW264 macrophages. Viability of GC-FA19 in RAW264 macrophages was visualized with BacLight staining. ***i***: GC-FA19 in medium alone incubated for 1 hr at room temperature prior to staining where red is dead and green is live GC-FA19. ***ii***: Uninfected RAW264 macrophages incubated at 37°C for 1 hr prior to staining in the presence of 0.1% saponin. ***iii***: RAW264 macrophages infected with GC-FA19 for 1 hr and stained without saponin. ***iv***: RAW264 macrophages infected with GC-FA19 for 1 hr and stained in the presence of 0.1% saponin. ***v***: RAW264 macrophages infected with GC-FA19 for 5 hr and stained in the presence of 0.1% saponin. These data are representative of three independent experiments.

In order to confirm the significance of our experimental findings generated using human monocytic cell lines THP-1 and MM6, we extended our work to primary peripheral human monocytes freshly obtained from healthy donors. We found that peripheral monocytes recognized the presence of live GC-FA19 infection and robustly responded by releasing cytokines IL-6 and TNFα as well as the antibacterial lipocalin NGAL (Figure 9A), and by upregulating hepcidin gene expression (Figure 9B). Further, we found that gonococcal infection in primary monocytes led to significant downregulation of ferroportin (Figure 9C) and BDH2 gene expression (Figure 9D). Since *in vivo* experimental models for GC are performed using primarily murine animal models [45], [46], we also investigated whether *N. gonorrhoeae* infection modulates iron-limiting innate immune defenses in murine RAW264 macrophages. Results similar to those in monocyte experiments were obtained; GC-FA19 infection induced nitric oxide release and upregulated murine HAMP1 (the hepcidin homologue), LCN2 (the NGAL homolog) and NRAMP1 expression from infected macrophages, while it downregulated expression of the murine BDH2 gene (Figure 10). The viability of adherent and intracellular GC-FA19 in RAW264 macrophages after 1 and 5 hr post infection was confirmed using bacterial live-dead staining (Figure 11). Notably, few macrophages were heavily infected with GC-FA19 after 5 hr of infection, which resembles the pattern of infected neutrophils observed in urethral exudates (Figure 11-*v*).

## Discussion

Human macrophages constitute the second wave of phagocyte influx that follows an often intense entry of polymorphonuclear leukocytes (PMNs) at sites of infection. While there is now compelling evidence that many gonococci survive this PMN influx and can exist within phagolysosomes, less is known about the fate of gonococci within macrophages. We evaluated the survival of gonococci in monocytes and macrophages and found that *N. gonorrhoeae* survived in association with these phagocytes and responded under the conditions employed to monitor phagocytic killing by upregulating a panel of its iron-responsive genes (*fetA, tbpA, tbpB, mpeR*) [34], [35], [36], [37]. Further, our findings indicate that some gonococci can survive within both human and murine macrophages. Importantly, our work revealed that such infected phagocytes could sense the presence of gonococci and respond robustly by secreting pro-inflammatory mediators. Hence, gonococcal stimulation of macrophages could have a profound influence on the continued expression of a pro-inflammatory, often damaging, response during natural infection.

Since gonococci can survive intraleukocytically [1], [5], [23], it is important to understand how they adapt to the intracellular environment and acquire nutrients within phagocytes for survival and growth. We asked if gonococci could manipulate the bioavailability of iron that is normally tightly regulated by macrophages. The capacity of gonococci to use TonB-dependent and -independent mechanisms of iron acquisition, either constitutively or induced upon infection, is considered essential for both intracellular and extracellular growth. Herein, we show that gonococci could facilitate iron acquisition by upregulating expression of some of its own iron-responsive genes and by modulating the host cellular iron metabolism, leading to increased intracellular labile iron pools in macrophages. In this respect, we found that infection of macrophages by live gonococci led to increased hepcidin gene and protein expression that would cause iron retention in the overall macrophage population. It is important to emphasize that this cationic peptide, unlike LL-37 (24 and data not presented), does not exert anti-gonococcal activity as assessed under the conditions of our *in vitro* assay used for other cationic peptides, which is consistent with hepcidin behaving more as a hormone as opposed to an antibacterial peptide [47]. Furthermore, while increased hepcidin-25 activity against other bacteria has been shown to be acid pH-dependent [39], [42], our data indicate that in the context of gonococci acidic pH does not increase the bactericidal activity of hepcidin-25. Therefore, when hepcidin concentration is increased in response to gonococcal infection in the acidic phagosome or the neutral pH cytosol of macrophages, gonococci would likely be able to survive this challenge. Others reported that hepcidin is induced in myeloid cells in response to bacterial pathogens via TLR4 [48] and bacterial cell wall components [49]. Further, gonococcal infection of macrophages also modulated the expression of other genes encoding factors involved in cellular iron metabolism. Specifically, its infection of macrophages reduced gene expression for ferroportin and BDH2 while increasing that for NRAMP1 and NGAL. Collectively, these changes would enhance the cytosolic iron pool in macrophages. Taken together, our data suggest that *N. gonorrhoeae* modulates the iron-limiting innate immune defenses in macrophages, which we propose would facilitate its ability to acquire iron and survive intracellularly.

Iron retention in mammalian cells has been reported to be associated with depletion of BDH2 [16], [17]. This enzyme is a homologue of EntA in *E. coli*, which mediates the synthesis of the bacterial siderophore 2,3-DHBA known as enterobactin [17]. Similarly, BDH2 mediates the synthesis of the mammalian siderophore 2,5-DHBA that binds and traffics labile cellular iron. BDH2 mRNA transcript contains an iron responsive element (IRE) that controls its expression in an iron-dependent manner, and has been shown to associate with cellular iron regulatory proteins (IRPs). Thus, BDH2 plays a very important role in cellular iron homeostasis [16]. To date, only the physiological role of BDH2 in cellular iron metabolism has been described. BDH2 depletion led to increased labile iron, which impacts mitochondrial iron-sulfur cluster biogenesis, heme synthesis and ROS redox balance [17]. Our study here is the first to report the role of BDH2 in innate immunity. Our findings suggest that BDH2 may be a new key player in the iron-limiting innate immune defenses against gonococcal infection in macrophages. We show that *N. gonorrhoeae* infection in macrophages resulted in significant downregulation of BDH2 expression. It is not known whether BDH2 downregulation is consequent to increased hepcidin expression since BDH2 harbors an IRE, and hence can be modulated by cellular iron retention [16]. The direct downregulation of BDH2 by gonococci cannot be ruled out and remains to be investigated. Nevertheless, the capacity of *N. gonorrhoeae* to modulate intracellular iron bioavailability by increasing hepcidin and reducing BDH2 expression is novel and may serve to facilitate gonococcal iron acquisition.

Other key components of cellular iron metabolism were also investigated in this study including NRAMP1, the endosomal iron transporter, and NGAL the iron carrier lipocalin. Interestingly, NRAMP1 plays an important role in host defense against intracellular bacterial infections such as tuberculosis by depleting iron from the phagolysosome where *Mycobacterium tuberculosis* (MTB) usually resides [50]. Genetic mutations/SNPs in NRAMP1 are shown to genetically predispose the host to infection with MTB and other intracellular pathogens [50]. Therefore, NRAMP1 is a key participant in iron metabolism and in host defense by exporting iron from the late endosome and phagolysosome, consequently increasing the transient labile cytosolic iron pool [51]. Importantly, we found that gonococcal infection of human monocytes or murine macrophages resulted in increased NRAMP1 gene expression, which is consistent with it being an integral part of the host defense to deplete iron in the phagolysosomal compartment. We also found that NGAL gene expression is highly upregulated in macrophages infected with gonococci. NGAL plays a role in both cellular iron homeostasis (since it shuttles liganded iron complexes such as the mammalian siderophore 2,5-DHBA [17], [52]) and in host defense (due to its ability to scavenge bacterial siderophores [19], [53]). NGAL is rapidly upregulated in response to infection and to other cellular stresses or perturbations. It has been shown that NGAL is highly upregulated during oxidative stress as an antioxidant cellular response [54]. NGAL also has adipokine function and therefore, exerts immunomodulatory effects on immune cells [55]. Gonococci are known to suppress adaptive and humoral immune responses [56]; therefore, the upregulation of NGAL may be an important part of the overall ability of this human pathogen to evade immune responses.

**Figure 12:**
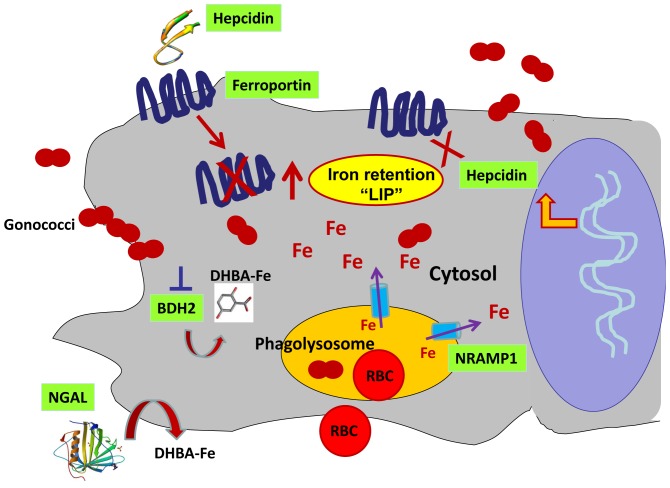
Model for how gonococcal infection in macrophages alters cellular iron homeostasis to facilitate iron acquisition. Macrophages play an essential role in iron homeostasis by engulfing senescent red blood cells (RBC) and recycling iron. Macrophages also play a very important role in host defense. GC infection in macrophages induces the expression of hepcidin (which then degrades the iron exporter ferroportin) and downregulates ferroportin gene expression, causing iron retention in macrophages. GC infection also induces expression of NRAMP1 (the cytosolic iron transporter) and NGAL (an iron carrier protein), and downregulates expression of BDH2, the enzyme that catalyzes the synthesis of the mammalian siderophore 2,5-DHBA which helps detoxify the labile iron pool (LIP). Collectively, these alterations in cellular iron homeostasis lead to increased iron bioavailability that facilitates iron acquisition and promotes gonococcal intracellular survival.

In summary, we report a novel mechanism by which *N. gonorrhoeae* modulates host cellular iron metabolism and innate immune defenses (Figure 12). We propose that this system facilitates gonococcal acquisition of iron so that it can survive within a macrophage. Given the longevity of macrophages and their capacity to migrate across epithelial surfaces, this survival strategy would potentiate the exacerbation and spread of infection.
